# Spiro’s portrait of Arnold Berliner—a testimonial to a deep friendship

**DOI:** 10.1007/s00114-023-01872-7

**Published:** 2023-09-02

**Authors:** Stefan L. Wolff

**Affiliations:** Forschungsinstitut des Deutschen Museums, München, Museumsinsel 1, 80538 Germany

In the former living room of Fritz Haber’s villa in Berlin-Dahlem, we can see a portrait created by the Breslau-born painter Eugen Spiro (1874–1972). Today, the room is used as a lecture hall of the Fritz-Haber-Institute, the former Kaiser-Wilhelm-Institute (KWI) for Physical Chemistry which was renamed in honor of its first director, the Nobel prize winner in chemistry who resigned in 1933 as a protest against the dismissals of the so-called non-Aryans in his staff.

Spiro was a well-known artist who created portraits of famous individuals from politics, business, culture, and science. In 1930, Otto Warburg, director of the KWI for Cell Physiology in Berlin-Dahlem, ordered portraits of his paradigm researchers Paul Ehrlich, Robert Koch, and Louis Pasteur, which adorned the largest room in his house, serving as his workspace and containing the library. The portraits are still there, in what is now the reading room of the archive of the Max Planck Society (Henning 2004, p.143; Henning and Kazemi 2009, p. 31). Spiro had already painted portraits of Fritz Haber and Max Planck in 1926 and 1928, respectively (Abercron 1990, p. 167 and p. 169; Uebele 2009, p. 276). Einstein agreed to such a portrait only in exile in 1941 (Abercron 1990, p. 64).

The painting in Haber’s villa depicts Arnold Berliner (1862–1942) (Fig. 1), the long-time editor of the journal “Die Naturwissenschaften” published by Springer-Verlag (Wolff 2023). This journal was also the official organ of the Kaiser Wilhelm Society since 1924. Below the painting, on the frame, is a nameplate with the life dates of Berliner. At the top right-hand corner of the painting, we see a dedication: “s. l. [seinem lieben: to my dear] Otto Jeidels z [zum: to]. 13.3.1926” (Fig 2). This date marked Otto Jeidels (1882–1947) 44^th^ birthday, for which Berliner had commissioned this painting as a gift. Different from the portraits mentioned above, it had a very intimate character showing him relaxed with a cigar in his hand. Berliner, who had a fondness for art and music, belonged to a group of patrons who supported Spiro. Berliner not only purchased paintings from Spiro but also financed his journey to Corsica in 1928 (Abercron 1990, p. 35; Spiro 2010, p. 9).

**Fig. 1:**
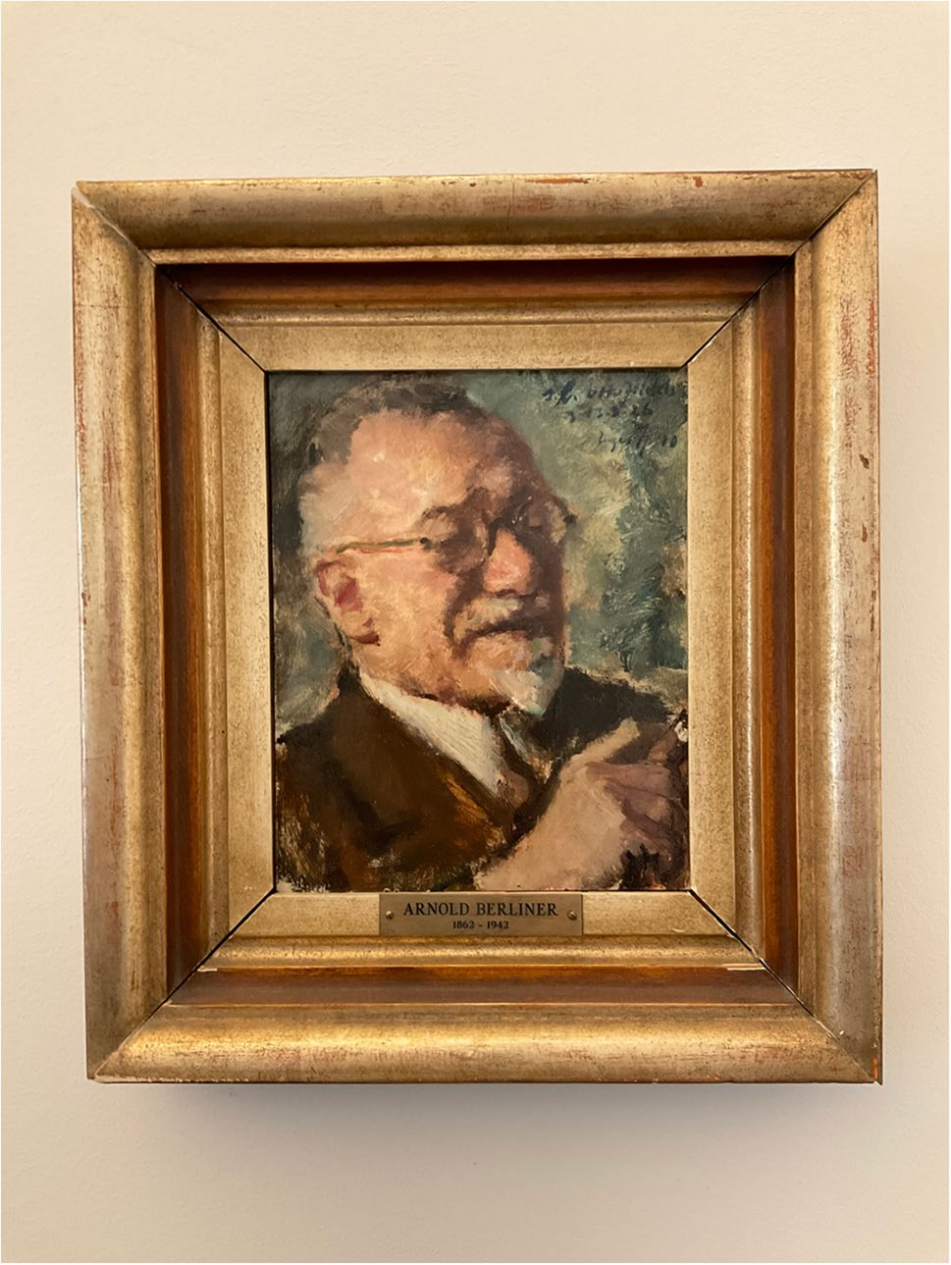
Spiro´s painting of Berliner, 1926 (photo: Albrecht Ropers)

**Fig. 2:**
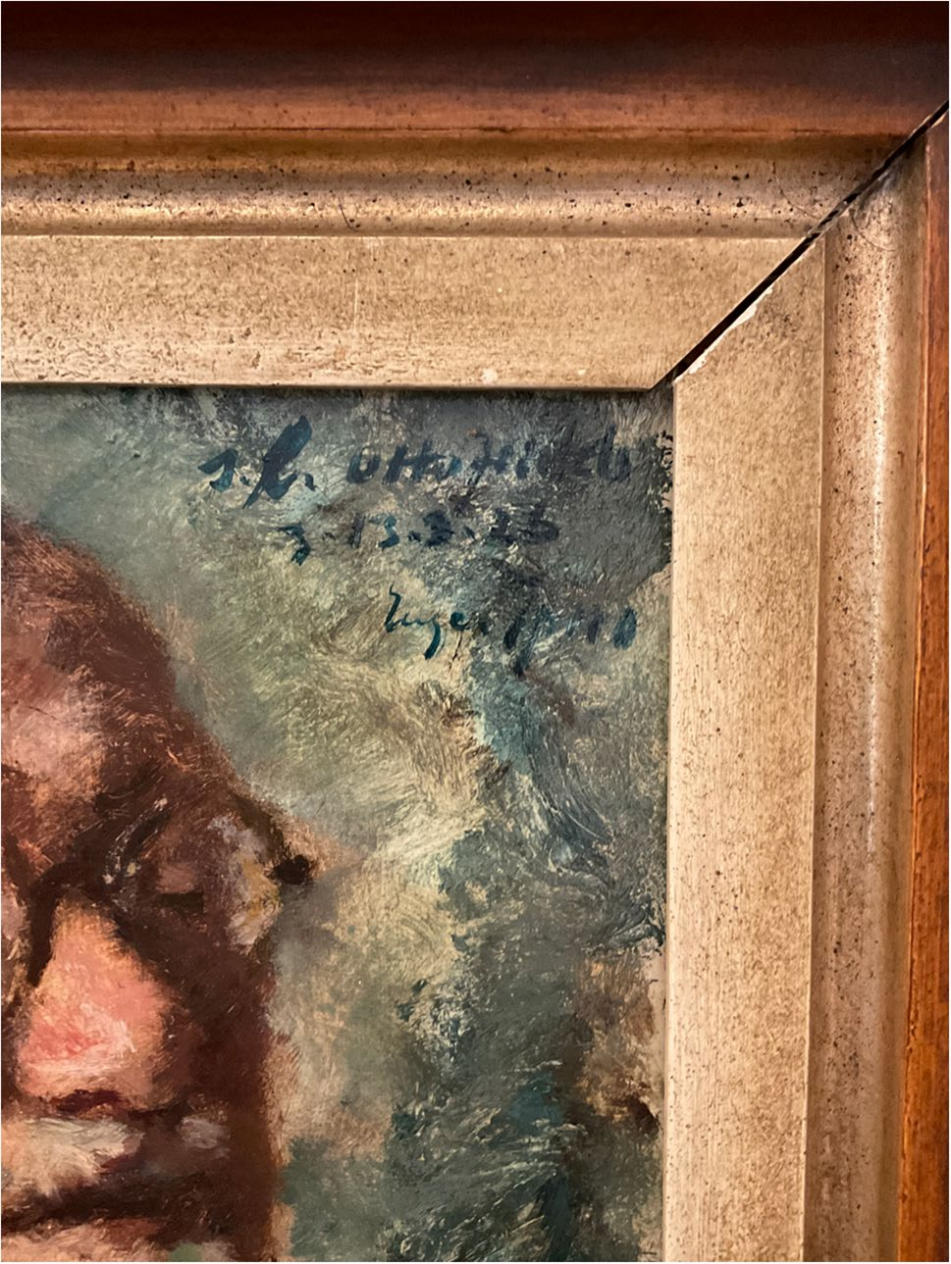
Dedication in Spiro´s painting (photo: Albrecht Ropers)

This painting is an expression of the deep friendship between Berliner and Jeidels, who was 20 years younger and descended from a wealthy Jewish business family from Frankfurt am Main. Jeidels wrote a dissertation on a topic of economic science in 1905 and, after spending some time abroad, worked for the “Berliner Handelsgesellschaft” since 1909, eventually becoming its managing director in 1918 (Jeidels 1905; Münzel and Kobrak 2011; Institut für Zeitgeschichte München 1980, p. 565).

Jeidels was one of the leading German bankers then (Fig. 3). He and his wife were also sponsors of Spiro (Abercron 1990, p. 36). As a patron, Jeidels became involved in the scientific community and got to know scientists. He was a member of the board of trustees of the “Einstein Foundation,” which provided funding for the Astrophysical Institute in Potsdam to investigate the experimental consequences of General Relativity (Goenner 2005, p. 169).^1^ While attending the board meetings, he received direct information about the state of research.^2^ These activities were often accompanied by social events, such as when Jeidels and his wife invited Einstein and the board members for dinner after such a meeting.^3^ Jeidels was one of the three sponsors who gifted Einstein a sail boat on the occasion of his 50th birthday in 1929.^4^

**Fig. 3:**
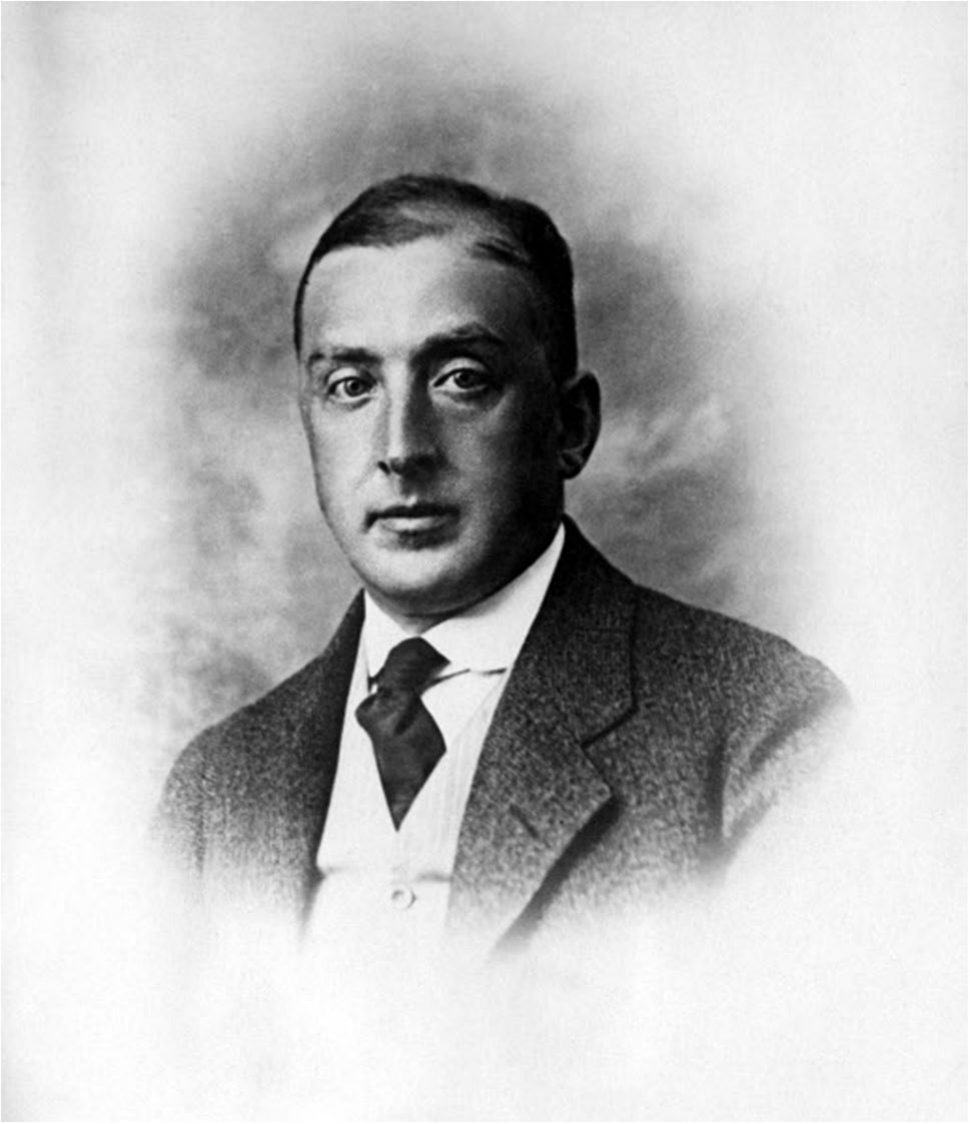
A portrait of Otto Jeidels, approx. 1928, bpk/Willy Römer: Otto Jeidels

The friendship between Berliner and Jeidels had already existed^5^ when they both became members of the “Deutsche Gesellschaft [von 1914]” during World War I (Deutsche Gesellschaft 1914, 1919, pp. 14 and 39). It was founded in 1915 as a club above party lines to preserve the spirit of “Burgfrieden.” This place of social interaction provided its members an exclusive space with an excellent library and an exquisite restaurant (Verhey 2000, pp. 163–166). After the war, Berliner and Jeidels together with Otto von Rottenburg, a consultant in the Prussian Ministry for Education, and Wolfgang Windelband, who conducted historical research on Bismarck, established an intellectual “round table” there. They saw each other almost daily at lunch and often invited foreign guests, including international known scholars (Ruge 1959, pp. 152–153; Flexner 1960, p. 229). Later, Berliner introduced his friend, the physicist and Nobel laureate Max von Laue (1879–1960), to this circle (Lemmerich 2011, p. 13). After the closure of the club in 1934, the “round table” found another place to continue for some years (Verhey 2000, p. 165).^6^ Jeidels chose the date of February 26, 1922, for his wedding, which coincided with Berliner’s 60th birthday. Berliner attended the ceremony as one of the two witnesses, making it a day of shared celebration.^7^

As an employee of the “Springer-Verlag,” Berliner, who had left Judaism in 1905,^8^ was not subject to the laws of April 1933 targeting “non-Aryan” public servants, which forced many scholars to emigrate. However, in 1935, Springer succumbed to pressure from the regime and suddenly dismissed Berliner from his position on August 13. He was even forbidden to enter the publishing house anymore (Sarkowski 1996, pp. 333–335).^9^ In September 1935, Berliner joined Laue on his tour to the US where Laue was invited to give talks which had been financed by Jeidels. Berliner hoped to find a new position in the US, and indeed, after a meeting in Princeton it looked as if an arrangement was possible. However, upon his return to Germany, it became evident that this could not be realized.^10^ In March 1938, Jeidels and his wife left Germany, emigrating to the US and taking Spiro’s painting of Berliner with them (Fig. 1). Berliner wrote to his friend Paul Epstein whom he had visited in his home in Pasadena during his last journey to the US in 1937: “It becomes more and more lonely around me, recently Dr. Jeidels also left – you may certainly remember him from the ‘Deutsche Gesellschaft’ – after 29 years of service at the ‘Berliner-Handels-Gesellschaft’, being a member of the directorate for 20 years. He was the closest to me among my acquaintances here. This is also a heavy blow for me.”^11^

In spring 1939, Jeidels met the émigré mathematician Richard Courant and the Danish physicist and Nobel laureate Niels Bohr in Princeton to discuss possibilities for getting Berliner out of Germany. However, they were unsuccessful.^12^

On February 12, 1942, the office of Albert Speer, who was the “General Building Inspector for the Reichshauptstadt Berlin” and responsible for the “Central Department for Resettlement” [of the Jewish population], sent an official from the foreign office to view Berliner’s flat. The visitor obviously expressed an interest in it which meant that Berliner would soon be evicted.^13^ Faced with the prospect of being forced out of his home, Berliner made the decision to end his life. He did so using hydrogen cyanide on the night of March 22 to 23, 1942.^14^ A few months later, Jeidels sent a notice to inform Einstein about Berliner’s death: “You have known Berliner well and he was devoted to you. … I have lost my dearest friend … He is the only man and friend in my past whom I have insistently and bitterly missed and I shall remain attached to his memory until my last hour.”^15^ When Niels Bohr came to the US again in 1944, Jeidels remembered Berliner: “Our mutual friend Arnold Berliner …has died as you have probably heard long ago. Among many acts of friendship over twenty years, I owed to him the first introduction into your outstanding scientific work and the earliest personal praise of your human qualities.”^16^ Jeidels himself passed away in 1947 during a vacation in Switzerland.^17^ In 1951, his widow gifted the portrait of Berliner to Max von Laue, who had been his friend for a long time. When Berliner did not leave his home during the last years, Laue visited him once every week until the day before his death. He was then director of the institute that would later bear Fritz Haber’s name. A lawyer who had been close to Berliner brought the painting from the US back to Berlin, and Laue thanked Jeidels’ widow: “I accept this precious gift with heartfelt thanks for the Kaiser-Wilhelm-Institute for Physical Chemistry in Berlin-Dahlem. It will have its place in the director’s office. It is a great pleasure to have my deceased friend in a picture in front of me in this way, particularly as there is hardly another picture of him. To the best of my knowledge, he was never photographed.”^18^

## Footnotes

1 Freundlich to Einstein 22.6.1922, Einstein Collected Papers, Vol. 13, 2012, Princeton University Press, p. 810.

2 Albert Einstein in Berlin, Part 1: Darstellung und Dokumente, 1979, Akademie-Verlag, Dokument Nr. 110, 6.12.1924: Sitzungsbericht des Kuratoriums der Einstein-Stiftung, p. 190.

3 Invitation to dinner after the meeting of 6.12.1924, note of 25.11.1924, Einstein Collected Papers, Vol. 14, 2015, Princeton University Press, p. 888.

4 Comment in: Einstein to Dora Stern 14.3.1929, Einstein Collected Papers, Vol. 16, 2021, Princeton University Press, p. 638.

5 Jeidels mentioned later that he had seen Berliner nearly daily for about 30 years: Jeidels to Einstein, 23.6.1942, AEA (Albert Einstein Archive) 7 049 The Hebrew University Jerusalem.

6 Laue to his son, 1.12.1940 (Lemmerich 2011, p. 185).

7 Mentioned in: Jeidels to Einstein, 23.6.1942, AEA 7 049 The Hebrew University Jerusalem.

8 Leaving Judaism on 7.8.1905, Archiv Centrum Judaicum Berlin, CJA, ZA1, Austrittskartei.

9 Laue to his son, 9.12.1945 (Lemmerich 2011, p. 283).

10 (Schmidt-Böcking et al. 2018, pp. 225–232), especially Jeidels to Courant, 18.2.1935, ibidem., p. 227. In his travel diary, Laue mentioned a meeting with Ware Cattell (1902–1969) in Princeton on 24.10.1935, where a cooperation with Berliner was discussed concerning a new “scientific encyclopedia,” planned by James McKeen Cattell (1860–1944), father of Ware Cattell and editor of Science. During the common return trip, which started on 22.11.1935, Laue added to a remark about an illness of Berliner: “He is far better than so far as he has a new occupation promised in the US.” Travel diary, Universitätsarchiv Frankfurt, Abt. 148 S4, No. 51, p. 25v and 46v.

11 Berliner to Epstein, 15.3.1938, Paul Sophus Epstein Papers, The Cal Tech Archives Pasadena.

12 Bohr to Richard Gregory, 9.6.1939, SPSL (Society for the Protection of Science and Learning) files 151 Hebrew University, p. 320–321, Bodeleian Library Oxford.

13 Berliner to Ruge, 13.12.1942, 4 Cod Ms. philos 220, Niedersächsische Staats- und Universitätsbibliothek Göttingen.

14 Otto Hahn believed that Berliner had still received this poison from Fritz Haber: Hahn, Beziehungen zu Nichtariern, England July 1945, Archiv MPG (Max-Planck-Gesellschaft) Abt. III, Rep. 14 A No. 6179. Transcribed and commented in: (Kaiser 2018, p. 254). This agrees with a statement from Rosbaud, according to (Sime 1996, pp. 473–474).

15 Jeidels to Einstein, 23.6.1942, AEA 7 049 The Hebrew University Jerusalem.

16 Jeidels to Bohr, 14.1.1944, General Correspondence Box 14, Folder 51, NBA (Niels Bohr Archive) Copenhagen.

17 Narration of the brother Hans Jeidels in H. Jeidels to Laue, 17.7.1947, MPGA II-50–964, p. 2–3.

18 Laue to Mrs. Jeidels, 29.10.1951, MPGA Rep. 50 No. 964, Berlin. However, Laue contradicts himself as in his letter to Meitner on 26.12.1942 he reported: “His housekeeper has sent me on older but very good photo on that occasion. He is sitting on his armchair, as usually till at last, keeping a book in his hands, while he is looking at the viewer.” (Lemmerich 1998, p. 244) This picture was not found anymore. But there exists at least one photo of Berliner.
